# Tobacco Product Use Among Youths With and Without Lifetime Asthma — Florida, 2016

**DOI:** 10.15585/mmwr.mm6721a2

**Published:** 2018-06-01

**Authors:** Keshia M. Reid, Jamie R. Forrest, Lauren Porter

**Affiliations:** 1 Florida Department of Health.

The increasing availability of diverse tobacco products has led to complex tobacco product use patterns among youths (*1*). Use by youths of products containing nicotine in any form is unsafe (*2*); among young persons with asthma, use of combustible tobacco products, particularly cigarettes, is associated with worsening symptoms, poor asthma control, and an increased need for medical management (*3*,*4*). Studies suggest that youths with asthma adopt health risk behaviors, including tobacco product use, at rates similar to or higher than those of youths without asthma (*3*–*7*); however, these studies are often limited to a partial list of tobacco product types among high school students. To assess current use (≥1 days during the past 30 days) of one or more of five tobacco product types (cigarettes, electronic cigarettes [defined as e-cigarettes, e-cigars, vape pipes, vaping pens, e-hookah, and hookah pens], hookah, smokeless tobacco, or cigars) among Florida middle school (grades 6–8) and high school (grades 9–12) students with or without a previous medical diagnosis of asthma, the Florida Department of Health analyzed data from the 2016 Florida Youth Tobacco Survey (FYTS). In 2016, 11.1% of middle school and 27.9% of high school students with asthma, and 7.9% of middle school and 24.2% of high school students without asthma, reported any current tobacco product use. Current use of each tobacco product type was considerably higher among students with asthma than among those without asthma. E-cigarettes were the most commonly used tobacco product type reported by middle and high school students with asthma (7.9% and 19.6%, respectively) and without asthma (5.8% and 17.2%, respectively). Statewide tobacco prevention strategies could help reduce all forms of tobacco product use among youths, particularly among those with asthma.

FYTS is a cross-sectional, school-based, pencil-and-paper questionnaire administered to Florida public middle school and high school students.* The consent process for the FYTS is dependent upon the school district administering the survey. Several districts use an active permission form; however, most districts obtain parental consent through passive permission forms that instruct parents to sign the form only if they wish to opt their child out of participation. Student participation is voluntary. Information is collected on indicators of tobacco product use, perceptions of harm, and exposure to secondhand smoke to monitor and guide tobacco prevention and control policies and strategies in Florida. A two-stage cluster probability sampling procedure was used to generate a statewide representative sample of Florida students attending public schools in grades 6–12. Data were self-reported, and no identifying information was collected from participating students. In 2016, a total of 36,082 middle and 33,558 high school students participated in the surveys; combined student and school response rates were 78.0% and 71.0%, respectively (*8*).

Lifetime asthma status was assessed with the question “Has a doctor or nurse ever told you that you have asthma?” Response options were “yes,” “no,” and “not sure.” Students who responded “yes” were categorized as having asthma; all other responses were categorized as not having asthma. Students who did not answer the question (9.8%) were excluded from analyses. Current use of tobacco products, defined as use on ≥1 days during the past 30 days, was measured for cigarettes, e-cigarettes,^†^ hookah (water pipes),^§^ smokeless tobacco products (chewing tobacco, snuff, or dip), and cigars (cigars, cigarillos, or little cigars).^¶^ “Any tobacco product use” was defined as current use of ≥1 tobacco products, and “multiple tobacco product use” was defined as current use of ≥2 products.

Data were statistically weighted to yield representative estimates of Florida middle and high school students attending public schools. Prevalence estimates and corresponding population counts were calculated for current tobacco product use overall and by school level (middle or high) among students with or without asthma. Rao-Scott modified chi-square tests were performed to assess statistically significant differences between students with or without asthma; p values of <0.05 were considered statistically significant.

In 2016, 19.5% of middle and 20.6% of high school students in Florida reported lifetime asthma (Table 1). Approximately 190,000 middle and high school students in Florida (8.5% and 24.9%, respectively) reported current use of any tobacco product in 2016, with approximately 86,000 (3.1% and 9.0%, respectively) reporting multiple tobacco product use.

**TABLE 1:** Prevalence of self-reported diagnosed asthma* among Florida middle and high school students, by school level ── Florida Youth Tobacco Survey, 2016

School level	Prevalence of asthma	
	%^†^ (95% CI)	Weighted no.^†^
**Total**	**20.1 (19.7–20.6)**	**261,786**
Middle school	19.5 (18.9–20.1)	157,468
High school	20.6 (20.0–21.2)	104,318

**Abbreviation:** CI = confidence interval.

* Asthma was defined as a “yes” response to the question “Has a doctor or nurse ever told you that you have asthma?”

^†^ Weighted to yield representative estimates of Florida middle and high school students attending public schools.

Current use of each tobacco product type was higher among middle and high school students with asthma than among those without asthma (p<0.05) (Table 2). Among students with asthma, 11.1% of middle (approximately 10,000) and 27.9% of high school (34,000) students reported current use of any tobacco product. Conversely, 7.9% of middle (30,000) and 24.2% of high school (116,000) students without asthma reported current use of any tobacco product. Among current users with asthma, 4.2% of middle (4,400) and 10.9% of high school (17,000) students reported multiple tobacco product use, compared with 2.8% of middle (12,000) and 8.6% of high school (52,000) students without asthma. During 2016, e-cigarettes were the most commonly used tobacco product type among middle (7.9%) and high school (19.6%) students with asthma, followed by hookah (3.8% and 9.7%), cigars (2.3% and 7.3%), cigarettes (2.2% and 5.7%), and smokeless tobacco (2.1% and 4.3%).

**TABLE 2:** Percentage of Florida middle and high school students who reported current tobacco product use,* by tobacco product, school level, and asthma status ── Florida Youth Tobacco Survey, 2016

Tobacco product	With asthma^†,§^		Without asthma		Total	
	%^¶^ (95% CI)	Weighted no. of users^¶^	%^¶^ (95% CI)	Weighted no. of users^¶^	%^¶^ (95% CI)	Weighted no. of users^¶^
**Middle school students**						
Cigarettes	2.2 (1.8–2.7)	2,273	1.4 (1.2–1.6)	5,845	**1.5 (1.4–1.7)**	**8,118**
Electronic cigarettes	7.9 (6.8–8.9)	7,202	5.8 (5.4–6.2)	22,554	**6.2 (5.8–6.6)**	**29,756**
Hookah	3.8 (3.1–4.5)	3,840	2.4 (2.1–2.6)	9,910	**2.7 (2.4–2.9)**	**13,750**
Smokeless tobacco	2.1 (1.6–2.5)	2,102	1.2 (1.0–1.3)	4,925	**1.3 (1.2–1.5)**	**7,028**
Cigars	2.3 (1.8–2.8)	2,385	1.3 (1.1–1.5)	5,654	**1.5 (1.3–1.7)**	**8,039**
Any tobacco product**	11.1 (9.3–12.3)	9,986	7.9 (7.4–8.4)	29,660	**8.5 (8.0–9.0)**	**39,646**
≥2 tobacco products^††^	4.2 (3.6–4.9)	4,426	2.8 (2.5–3.1)	12,146	**3.1 (2.8–3.4)**	**16,572**
**High school students**						
Cigarettes	5.7 (5.0–6.4)	8,663	4.6 (4.3–5.0)	27,520	**4.8 (4.5–5.2)**	**36,183**
Electronic cigarettes	19.6 (18.1–21.2)	22,979	17.2 (16.4–18.0)	80,412	**17.7 (17.0–18.4)**	**103,391**
Hookah	9.7 (8.6–10.7)	14,886	6.8 (6.3–7.2)	40,342	**7.4 (6.9–7.8)**	**55,227**
Smokeless tobacco	4.3 (3.7–4.9)	6,580	3.3 (3.0–3.6)	19,387	**3.5 (3.2–3.8)**	**25,967**
Cigars	7.3 (6.5–8.2)	11,187	5.5 (5.1–5.9)	32,874	**5.9 (5.5–6.3)**	**44,061**
Any tobacco product**	27.9 (26.2–29.6)	34,358	24.2 (23.3–25.1)	116,070	**24.9 (24.1–25.8)**	**150,428**
≥2 tobacco products^††^	10.9 (9.8–11.9)	17,114	8.6 (8.1–9.1)	52,057	**9.0 (8.6–9.5)**	**69,171**

**Abbreviation:** CI = confidence interval.

* Current cigarette use was defined as having smoked cigarettes on ≥1 days during the past 30 days; current electronic cigarette use was defined as having used an electronic cigarette on ≥1 days during the past 30 days; current hookah use was defined as having smoked tobacco from a hookah or water pipe on ≥1 days during the past 30 days; current smokeless tobacco use was defined as having used chewing tobacco, snuff, or dip on ≥1 days during the past 30 days; current cigar use was defined as having smoked cigars, cigarillos, or little cigars on ≥1 days during the past 30 days.

^†^ Having asthma was defined as a “yes” response to the question “Has a doctor or nurse ever told you that you have asthma?”

^§^ Use of each tobacco product type significantly differed by asthma status: Rao-Scott chi-square test; p value <0.05.

^¶^ Weighted to yield representative estimates of Florida middle and high school students attending public schools.

** Any tobacco product use was defined as having used cigarettes, electronic cigarettes, hookah, smokeless tobacco, or cigars on ≥1 days during the past 30 days.

^††^ ≥2 tobacco product use was defined as having used two or more tobacco products (cigarettes, electronic cigarettes, hookah, smokeless tobacco, or cigars) on ≥1 days during the past 30 days.

## Discussion

In 2016, Florida middle and high school students with asthma reported higher current use of each tobacco product type, including use of multiple tobacco products, than did students without asthma. The higher use of each tobacco product type among students with asthma, most notably combustible products (e.g., cigarettes), is concerning because health risks associated with tobacco product use are higher for this population (*3*,*4*).

Among middle and high school students with asthma who reported current use of tobacco products, e-cigarettes were the most commonly reported tobacco product type used, followed by hookah, cigars, cigarettes, and smokeless tobacco. Higher current use of all tobacco product types among youths with asthma might be explained by some of the same factors associated with cigarette smoking in this population, including exposure to tobacco product use in the social environment and use as a coping mechanism for psychosocial distress (*9*,*10*). Youths with asthma who are nonadherent to their asthma medication regimen also report being more rebellious and risk-taking, making them more likely to engage in health-compromising behaviors (*10*). Finally, high prevalence of e-cigarette and hookah use might reflect perceptions among youths that these products are more socially acceptable or less harmful than cigarettes, cigars, and smokeless tobacco (*6*,*7*). However, the use of products containing nicotine in any form among youths, including e-cigarettes, is unsafe (*2*).

The findings in this report are subject to at least four limitations. First, FYTS data are only collected from middle and high school students who attend public schools and are therefore not representative of all students or youths in Florida. Second, FYTS data are self-reported and therefore subject to recall and response bias. Third, past-30-day use of tobacco products might not reflect regular or daily use. Finally, because of questionnaire wording, it was not possible to ascertain the proportion of e-cigarette users who were vaporizing liquids containing nicotine versus other substances.

Statewide tobacco prevention strategies, particularly among youths with asthma, coupled with other proven interventions, are important to reducing all forms of tobacco product use among youths. As the diversity of tobacco products increases, measures to educate youths about the health risks for all tobacco product types are warranted, particularly among those with asthma.

**Summary**

**What is already known about this topic?**

Among young persons with asthma, use of combustible tobacco products, particularly cigarettes, worsens symptoms and increases need for medical management.

**What is added by this report?**

Current use of all tobacco product types and multiple tobacco products was significantly higher among students with asthma than among those without asthma. E-cigarettes were the most commonly reported tobacco product type used by middle and high school students with asthma (7.9% and 19.6%, respectively) and without asthma (5.8% and 17.2%, respectively).

**What are the implications for public health practice?**

Statewide tobacco prevention strategies could help reduce all forms of tobacco product use among youths, particularly among those with asthma.

## Footnotes

* FYTS is administered annually at the beginning of each calendar year. In even-numbered years, samples are drawn at the county level. http://www.floridahealth.gov/statistics-and-data/survey-data/florida-youth-survey/florida-youth-tobacco-survey/index.html.

† Electronic cigarettes are battery-operated devices that usually contain a nicotine-based liquid that is vaporized and inhaled. Questions about electronic cigarettes on FYTS were preceded by an introductory paragraph. This paragraph read “The next questions include e-cigarettes, e-cigars, vape pipes, vaping pens, e-hookah, and hookah pens made by companies such as Blu, NJOY, and Starbuzz.”

§ Hookah was defined by FYTS as a single-stemmed water pipe used for smoking tobacco.

¶ Current cigarette use was defined as having smoked cigarettes on ≥1 days during the past 30 days; current electronic cigarette use was defined as having used an e-cigarette, e-cigar, vape pipe, vaping pen, e-hookah, or hookah pen on ≥1 days during the past 30 days; current hookah use was defined as having smoked tobacco from a hookah or water pipe on ≥1 days during the past 30 days; current smokeless tobacco use was defined as having used chewing tobacco, snuff, or dip on ≥1 days during the past 30 days; current cigar use was defined as having smoked cigars, cigarillos, or little cigars on ≥1 days during the past 30 days.
